# Supplementary material for: Femtosecond time synchronization of optical clocks off of a flying quadcopter
Source: Nat Commun. 2019 Apr 18;10:1819. doi: 10.1038/s41467-019-09768-9 (PMC6472402; doi:10.1038/s41467-019-09768-9)
Supplement: Supplementary file 1 — Supplementary Information [file 41467_2019_9768_MOESM1_ESM.docx]

Supplementary Video 1 shows synchronization via a quadcopter-mounted retroreflector. The quadcopter is flow along the line-of-sight of the path with closing velocities which reach 20 m/s. The timing signals necessary for synchronization of the two clocks traverse the bi-directional link to this quadcopter. A graphical display indicates the relative time of the two clocks, the round trip optical pathlength, the closing velocity, whether the clocks are actively synchronized, and the percent of the time over a 1-second window without signal fades i.e. when the optical link is not lost due to tracking terminal mis-alignment or turbulence-induced fades.
